# Does statin use affect amyloid beta deposition and brain metabolism?

**DOI:** 10.1111/cns.14117

**Published:** 2023-02-14

**Authors:** Fardin Nabizadeh, Parya Valizadeh, Mohammad Balabandian, Alzheimer’s disease Neuroimaging Initiative (ADNI)

**Affiliations:** ^1^ Neuroscience Research Group (NRG) Universal Scientific Education and Research Network (USERN) Tehran Iran; ^2^ School of Medicine Iran University of Medical Sciences Tehran Iran; ^3^ School of Medicine Tehran University of Medical Science Tehran Iran

**Keywords:** Alzheimer's disease, amyloid beta, cognitive impairment, metabolism, statins

## Abstract

**Background:**

There are contradictory findings regarding the effect of statin drugs on amyloid β (Aβ) deposition as one of the main hallmarks of Alzheimer's disease (AD), along with tau pathology. We aimed to longitudinally investigate the therapeutic and preventive role of statin drugs by examining the brain Aβ deposition and metabolism rate in AD, mild cognitive impairment (MCI), and healthy controls (HC).

**Methods:**

The data of 828 subjects including 178 HC, 492 MCI, and 158 AD individuals were obtained from ADNI. The baseline and longitudinal [^18^F] AV45 and 18‐fluorodeoxyglucose (FDG) PET standard uptake value ratio (SUVR) measures were investigated among statin users and non‐users.

**Results:**

Our results showed that there is no significant difference in baseline Aβ deposition and metabolism rate between statin users and non‐users among HC, MCI, and AD subjects. While there was no significant effect of statin on metabolism rate, there was a significant difference in Aβ deposition change after 4 years (from baseline) between statin users and non‐users within HC subjects (*p* = 0.011). The change of Aβ deposition at 4 years from baseline was −2.0 ± 6.3% for statin users and 1.4 ± 4.7% for non‐users. There was no significant association between statin duration use with baseline and longitudinal Aβ deposition and metabolism rate. However, statin dosage was significantly associated with Aβ deposition in 2 years (*r* = −0.412, *p* = 0.021) in the HC group. Moreover, our analysis showed a significant correlation between total statin exposure (duration×dosage) and Aβ deposition in 2 years visit (*r* = −0.198, *p* = 0.037) in HC subjects. Furthermore, we investigated the longitudinal changes within each group of statin users and non‐users separately in linear mixed models. Our findings showed that there are no significant changes in AV45 and FDG SUVR among both groups.

**Conclusion:**

The present longitudinal analysis revealed that using statins might be beneficial in slowing down or stabilizing the Aβ deposition due to aging in subjects without cognitive impairment. However, once the clinical symptoms of cognitive impairment appear, statins fail to slow down Aβ deposition. Overall, our findings revealed that statin users might have slower Aβ aggregation than non‐users.

## Graphical abstract

Representation of AV45 (A), and FDG SUVR (B) levels at different time points. Our results showed that there is no significant difference in baseline Aβ deposition and metabolism rate between statin users and non‐users among HC, MCI, and AD subjects. While there was no significant effect of statin on the metabolism rate, there was a significant difference in Aβ deposition change after 4 years (from baseline) between statin users and non‐users within HC subjects (*p* = 0.011).

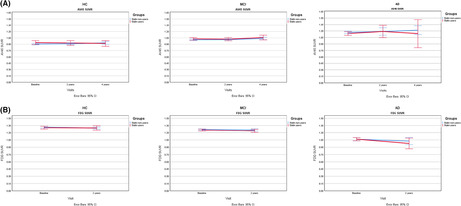

## 1 INTRODUCTION

Alzheimer's disease (AD) is an aggressive and progressive neurodegenerative disease known as the primary cause of dementia.^1^ The first presentation of AD is memory loss in the vast majority of cases with other symptoms such as cognitive dysfunction, psychiatric symptoms, and behavioral disturbances.^1^ Although the exact etiology of AD is yet to be known, it is believed that two factors play a crucial role in AD pathology: amyloid β (Aβ) plaques and tau tangles.^1^ Moreover, reduced cerebral blood flow (CBF) and neurovascular dysfunction associated with Aβ plaques are major contributions to the progress of AD.^2^ Elevated levels of Aβ in the brain are significantly associated with cognitive decline, and specific regions of the brain elevate even before the occurrence of tau pathology, the other pathological hallmark of AD.^3^ Besides Aβ plaques and tau neurofibrillary tangles, glucose hypometabolism is also a part of the pathological mechanism of AD and could be used as a diagnostic factor in preclinical stages.^4^ Along with Aβ deposition, cerebral hypometabolism assessed by fluorodeoxyglucose PET (FDG‐PET) is significantly correlated with AD biomarkers along disease progression.^5^

3‐hydroxy‐3‐methylglutaryl coenzyme A reductase inhibitor (HMG‐CoA inhibitor) also known as statins are lipid‐lowering agents (LLAs) used in lowering low‐density lipoprotein (LDL) cholesterol.^6^ Statins are the most widespread LLAs used for primary and secondary prevention of cardiovascular events.^7^ For many years, there were reports on the impact of these drugs on neurological and psychiatric disorders. Statins could significantly reduce depression and hospitalization in patients prone to psychiatric disorders.^8^ Moreover, based on several studies, use of statin is correlated with less severe symptoms in patients with neurodegenerative diseases such as AD and Parkinson's disease (PD).^9^

There have been reports on the probable effect of statins on future AD development. Recently, a meta‐analysis conducted by Zhang et al. concluded that statins, by inhibiting the formation of intracerebral amyloid, have a protective role against dementia and AD.^10^ However, preventing cognitive decline may not be noticeable in short follow‐ups among AD patients. In line with these findings, another study also suggested that statins have no contributions to dementia but may need additional investigations before using them in aggressive lipoprotein therapies in elderly people.^11^

Previous studies showed the alternated cholesterol profile in AD patients and suggested that statins could be used as an efficacious therapy in AD.^12^ Different epidemiological studies indicated that statin use can be preventive and reduce the risk of AD and regulates the Aβ and possibly tau metabolism.^13^ Due to the contradictory findings regarding the role of statins in prevention, treatment, or even worsening AD, there is a strong need for further investigation to examine the effect of statins on AD development not only epidemiologically.^10^, ^14^, ^15^ Therefore, we aimed to longitudinally investigate the therapeutic and preventive role of statin drugs by examining the brain Aβ deposition and metabolism rate in AD, mild cognitive impairment (MCI), and healthy controls (HC).

## 2 MATERIALS AND METHODS

### 2.1 Subjects

Data were obtained from the Alzheimer's Disease Neuroimaging Initiative (ADNI) database (adni.loni.usc.edu). The ADNI was established in 2003 as a public–private partnership led by the Principal Investigator Michael W. Weiner, MD. The main purpose of ADNI is to test whether serial magnetic resonance imaging (MRI), positron emission tomography (PET), other biological markers, and clinical and neuropsychological assessments can be used to track the development of MCI and early AD.

**FIGURE 1:**
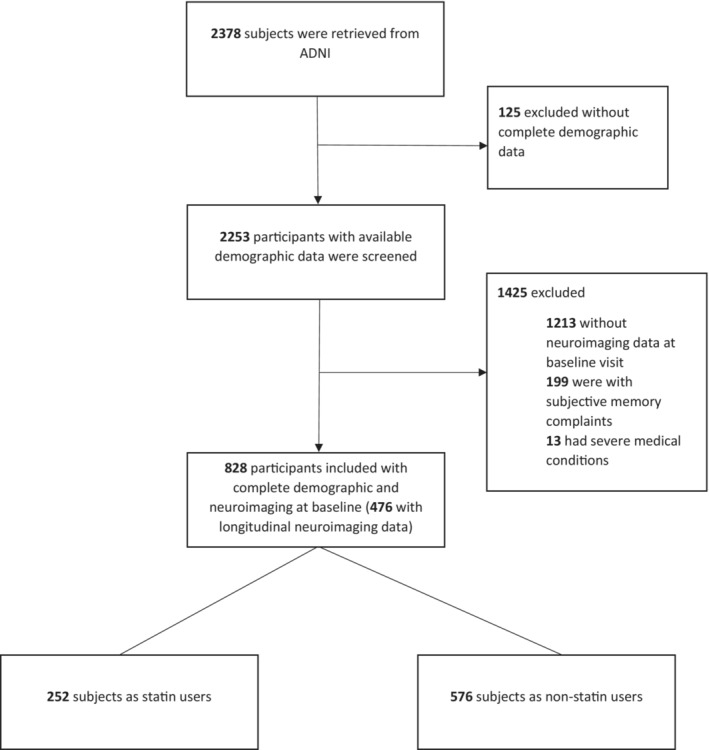
Flow diagram of study enrollment.

Alzheimer's disease Neuroimaging Initiative (ADNI2, ADNI3, and ADNIGO) participants who had baseline [^18^F]AV45 and 18‐fluorodeoxyglucose (FDG) PET measures and also available demographic information were initially selected (Figure 1, Table 1). Subjects with subjective memory concerns (SMC) due to the low number were excluded. Finally, the participants in the following groups were entered into our study: HC (*n* = 178), MCI (*n* = 492), and AD (*n* = 158).

**TABLE 1:** Number of participants with available PET data at each time points.

Variable	HC (*n* = 178)	MCI (*n* = 492)	AD (*n* = 158)
AV45 SUVRat baseline (number of statin users)	178 (53)	492 (146)	158 (63)
AV45 SUVR at 2 years (number of statin users)	134 (41)	311 (93)	31 (11)
AV45 SUVR at 4 years (number of statin users)	81 (30)	151 (47)	17 (4)
FDG SUVR at baseline (number of statin users)	178 (53)	492 (146)	158 (63)
FDG SUVR change at 2 years (number of statin users)	116 (39)	214 (58)	26 (7)

Abbreviations: AD, Alzheimer's disease; HC, healthy controls; MCI, mild cognitive impairment; SUVR, standard uptake value ratios.

All MCI subjects were diagnosed with amnestic MCI based on the following criteria: this diagnostic classification required Mini‐Mental State Examination (MMSE) scores between 24 and 30, a memory complaint, objective memory loss measured by education‐adjusted scores on the Wechsler Memory Scale Logical Memory II, a Clinical Dementia Rating (CDR) of 0.5, absence of significant impairment in other cognitive domains, essentially preserved activities of daily living, and absence of dementia. The AD ADNI subjects were also diagnosed according to the National Institute of Neurological and Communicative Disorders and Stroke–Alzheimer's Disease and Related Disorders Association (NINCDS‐ADRDA) criteria for probable AD and have MMSE scores between 20 and 26 (inclusive) and CDR of 0.5 or 1.

### 2.2 Classification of statin exposure

Information on statin use was obtained from the ADNI concurrent medication file, which is a record of the longitudinal use of medications among participants. The subjects with at least 2 years of statin use with a minimum of 10 mg per day were categorized as statin users. The duration of statin use was estimated by subtracting the date of the baseline visit date and the start date.

### 2.3 PET imaging biomarkers

Aβ deposition was visualized with PET tracer [^18^F]AV45.^16^ Measures of regional AV45 standard uptake value ratios (SUVR) which were calculated by ADNI core (Jagust Lab, UC Berkeley) were obtained (adni.loni.usc.edu). To calculate global Aβ deposition, the mean uptake in prefrontal, orbitofrontal, parietal, temporal, anterior cingulate, and posterior cingulate/precuneus regions of interest (ROIs) is standardized by dividing on the cerebellum and composite reference region for cross‐sectional and longitudinal analysis, respectively.^17^ The AV45 SUVR at different time points (baseline, 2, and 4 years) were obtained and entered into our analyses.

Data of the cerebral metabolic rate measured by FDG PET at baseline and 2‐year visits were retrieved from the ADNI server (Jagust Lab, UC Berkeley). The image processing techniques were previously described.^18^ FDG SUVR was defined by averaging FDG uptake in the angular gyrus, inferior temporal gyrus, and posterior cingulate, which were identified frequently in the previous investigations divided on top 50% FDG uptake of pons and cerebellum reference region.^19^, ^20^ The reference regions (top 50% pons and cerebellum) were used to decrease nuisance variability in FDG uptake between participants.

### 2.4 Cognitive assessments

All participants underwent Mini‐Mental State Examination (MMSE), which included 30 questions to measure the cognitive status at baseline. The MMSE score of patients was downloaded from ADNI.

### 2.5 APOE ε4 genotyping

The data of APOE ε4 genotyping of the participants were extracted from the ADNI dataset. The participants were divided into APOE ε4 positive and negative.

### 2.6 Statistical analysis

We used SPSS version 22 (BM Corp., Armonk, NY, USA) for statistical analysis. The Kolmogorov–Smirnov test was used to check the normality of the data. For comparison of the demographical and clinical characteristics of the statin users and non‐users, we used the t‐test and Mann–Whitney *U*‐test for parametric and non‐parametric variables, respectively. In order to assess the association between statin exposure and baseline PET findings, the ANCOVA models were used. Furthermore, to measure the effect of statin exposure on longitudinal neuroimaging findings, the PET measures were expressed as percent change from baseline at 2 and 4 years, and ANCOVA models were used to examine the difference between statin users and non‐users at each time point. To investigate the longitudinal changes of PET measures within each group, we used linear mixed models. To measure the association between the duration of statin use and dosage with baseline and longitudinal PET findings, multivariable linear regression models were used adjusted for the effect of statin types. All ANCOVA and regression models were adjusted for the effect of age, sex, APOE ε4 genotyping, MMSE score, cardiovascular conditions (hypertension, diabetes, hypercholesterolemia, coronary artery disease, and dyslipidemia), and cholinesterase drug use based on previous studies. A *p*‐value < 0.05 is considered statistically significant. The whole statistical process was performed separately in HC, MCI, and AD subjects. We used the Benjamini–Hochberg method to address the Type I error due to the multiple comparisons.

## 3 RESULTS

### 3.1 Participant's characteristics

Overall, 30.4% of our subjects were statin users. The mean duration of statin use was 6.5 ± 5.7, and 67% of statin users received simvastatin followed by pravastatin (13%). The demographic and clinical characteristics of the subjects are detailed in Table 2.

**TABLE 2:** Participants characteristics.

Characteristic	Group	Statin users (*n* = 252)	Statin non‐users (*n* = 576)	*p* Value
Number	HC	43	135	‐
	MCI	146	346	‐
	AD	63	95	‐
Age, mean (SD), years	HC	72.9 (6.2)	73.2 (6.4)	0.797
	MCI	72.7 (6.8)	71.1 (7.6)	0.03
	AD	75.0 (7.4)	73.9 (8.9)	0.422
Female sex, No. (%)	HC	22 (51.2)	70 (51.9)	0.938
	MCI	64 (43.8)	155 (44.8)	0.845
	AD	21 (33.3)	47 (49.5)	0.045
Education, mean (SD), years	HC	16.7 (2.4)	16.4 (2.5)	0.522
	MCI	16.2 (2.6)	16.2 (2.6)	0.984
	AD	15.8 (2.3)	15.5 (2.7)	0.584
MMSE score, mean (SD)	HC	27.9 (2.4)	28.8 (1.4)	0.008
	MCI	26.9 (3.1)	26.7 (4.1)	0.6
	AD	21.2 (4.3)	20.8 (3.8)	0.635
APOE‐e4 positive, No. (%)	HC	14 (32.6)	36 (26.7)	0.457
	MCI	74 (50.7)	160 (46.2)	0.286
	AD	40 (63.5)	62 (65.3)	0.854
Statin use duration (until baseline visit), mean (SD), years	HC	6.4 (5.2)	‐	‐
	MCI	6.6 (4.9)	‐	‐
	AD	6.4 (4.4)	‐	‐
Cardiovascular conditions, No. (%)	HC	19 (44.2)	42 (31.1)	0.117
	MCI	47 (32.2)	67 (19.4)	0.002
	AD	9 (14.3)	2 (2.1)	0.003

*Note*: Cardiovascular conditions included hypertension, diabetes, hypercholesterolemia, coronary artery disease, and dyslipidemia.

Abbreviations: AD, Alzheimer's disease; APOE, apolipoprotein E; HC, healthy controls; MCI, mild cognitive impairment; MMSE, Mini‐Mental State Exam.

A comparison between statin users and non‐users showed HC subjects who did not receive statin had higher MMSE scores at baseline (*p* = 0.008). Moreover, statin users among MCI and AD patients had a higher prevalence of cardiovascular conditions (hypertension, diabetes, hypercholesterolemia, coronary artery disease, and dyslipidemia) compared to non‐users (*p* = 0.002, *p* = 0.003).

### 3.2 Effect of statin on PET findings

We initially investigated the association between statin use and baseline AV45 and FDG SUVR using ANCOVA models. Our results showed that there is no significant difference in baseline Aβ deposition and metabolism rate between statin users and non‐users among HC, MCI, and AD subjects (Table 3).

**TABLE 3:** Brain PET changes in statin users and non‐users within ANCOVA models.

Variable	Statin users (*n* = 252)	Statin non‐users (*n* = 576)	*p* Value
HC			
AV45 SUVRat baseline, mean (SD)	1.37 (0.27)	1.31 (0.19)	0.511
AV45 SUVR change at 2 years (from baseline), % (SD)	−0.5 (4.5)	1.3 (2.9)	0.093
AV45 SUVR change at 4 years (from baseline), % (SD)	−2.0 (6.3)	1.4 (4.7)	0.011
AV45 SUVR change at 4 years (from 2 years), % (SD)	−2.1 (6.9)	0.1 (3.8)	0.014
FDG SUVR at baseline, mean (SD)	1.30 (0.09)	1.31 (0.11)	0.454
FDG SUVR change at 2 years (from baseline), % (SD)	−0.3 (5.0)	−1.5 (4.3)	0.149
MCI			
AV45 SUVRat baseline, mean (SD)	1.49 (0.29)	1.43 (0.27)	0.364
AV45 SUVR change at 2 years (from baseline), % (SD)	1.6 (3.7)	3.9 (15.9)	0.168
AV45 SUVR change at 4 years (from baseline), % (SD)	2.6 (4.8)	3.6 (12.4)	0.491
AV45 SUVR change at 4 years (from 2 years), % (SD)	0.9 (4.2)	0.3 (4.3)	0.592
FDG SUVR at baseline, mean (SD)	1.25 (0.13)	1.26 (0.13)	0.642
FDG SUVR change at 2 years (from baseline), % (SD)	−2.5 (5.7)	−2.1 (5.8)	0.701
AD			
AV45 SUVRat baseline, mean (SD)	1.62 (0.27)	1.63 (0.25)	0.698
AV45 SUVR change at 2 years (from baseline), % (SD)	2.8 (3.0)	3.6 (6.6)	0.758
AV45 SUVR change at 4 years (from baseline), % (SD)	−0.7 (4.2)	6.1 (4.4)	0.344
AV45 SUVR change at 4 years (from 2 years), % (SD)	−2.6 (1.5)	3.7 (4.0)	0.330
FDG SUVR at baseline, mean (SD)	1.06 (0.12)	1.06 (0.15)	0.812
FDG SUVR change at 2 years (from baseline), % (SD)	−9.6 (7.4)	−7.0 (5.6)	0.799

*Note*: *P* value as defined using ANCOVA models adjusted for age, sex, APOE e4, MMSE score, cardiovascular conditions, and cholinesterase drugs use.

Abbreviations: AD, Alzheimer's disease; HC, healthy controls; MCI, mild cognitive impairment; SUVR, standard uptake value ratios.

**FIGURE 2:**
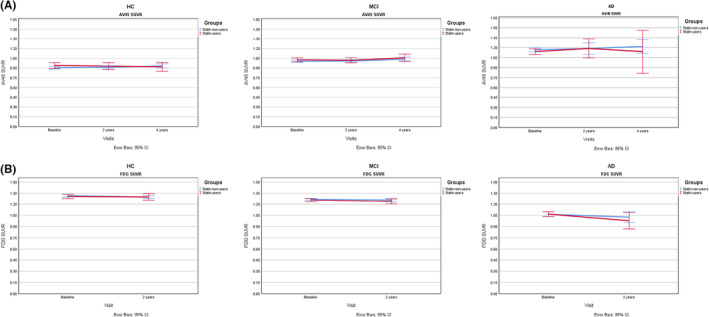
Representation of AV45 (A), and FDG SUVR (B) levels at different time points.

Furthermore, to assess the effect of statin use on longitudinal Aβ deposition and metabolism rate, we compared the change at 2 and 4 years (Figure 2). The percent change of FDG SUVR at 2 years in HC subjects were −0.3 ± 5.0% and −1.5 ± 4.3% for statin users and non‐users, respectively (*p* = 0.149). The change of AV45 SUVR at 2 years was −0.5 ± 4.5% for statin users and 1.3 ± 2.9% for non‐users among the HC group (*p* = 0.093). While there was no significant effect of statin on metabolism rate, there was a significant difference in AV45 SUVR change at 4 years (from baseline) between statin users and non‐users within HC subjects (*p* = 0.011). The change of AV45 SUVR at 4 years from baseline was −2.0 ± 6.3% for statin users and 1.4 ± 4.7% for non‐users. Moreover, there was also a significant difference in AV45 SUVR change at 4 years from 2 years visit between statin users and non‐users of the HC group (*p* = 0.014). The change was −2.1 ± 6.9% and 0.1 ± 3.8% for statin users and non‐users, respectively.

In MCI patients, the percent change of FDG SUVR at 2 years from baseline was −2.5 ± 5.7 for statin users and −2.1 ± 5.8 for non‐users (*p* = 0.701). At 2 years, change in AV45 SUVR from baseline was 1.6 ± 3.7% for statin users and 3.9 ± 15.9% for non‐users (*p* = 0.168). Also, the AV56 SUVR change at 4 years from baseline and 2‐year visits was 2.6 ± 4.8% and 0.9 ± 4.2% for statin users and 3.6 ± 12.4% and 0.3 ± 4.3% for non‐users (*p* = 0.491, 0.592).

There was no difference in FDG SUVR change at 2 years between statin users (−9.6 ± 7.4%) and non‐users (−7.0 ± 5.6%) in AD patients (*p* = 0.799). Furthermore, in the AD group, we found that there was no significant difference in change of AV45 SUVR at 2 and 4 years from baseline and at 4 years from 2 years visits between statin users and non‐users. The AV45 SUVR change is represented in Table 3.

Furthermore, we investigated the longitudinal changes within each group of statin users and non‐users separately in linear mixed models (Table 4). Our findings showed that there are no significant changes in AV45 and FDG SUVR among both groups.

**TABLE 4:** Longitudinal changes of brain PET changes in statin users and non‐users within linear mixed models.

Variable	Statin users (*n* = 252)	Statin non‐users (*n* = 576)
HC		
AV45 SUVR change at 2 years	0.615	0.134
AV45 SUVR change at 4 years	0.791	0.294
FDG SUVR change at 2 years	0.773	0.694
MCI		
AV45 SUVR change at 2 years	0.312	0.142
AV45 SUVR change at 4 years	0.26	0.092
FDG SUVR change at 2 years	0.476	0.512
AD		
AV45 SUVR change at 2 years	0.998	0.585
AV45 SUVR change at 4 years	0.643	0.75
FDG SUVR change at 2 years	0.131	0.178

*Note*: *p*‐Values are presented. Significant results are bolded.

Abbreviations: AD, Alzheimer's disease; HC, healthy controls; MCI, mild cognitive impairment; SUVR, standard uptake value ratios Longitudinal changes in the liner mixed model within each group at each time point from baseline.

Further analysis was done to see whether there is a systematic bias among participants with longer follow‐ups. We investigated the difference of AV45 and FDG SUVR in subjects with only 2 years of follow‐up versus subjects with 4 years of follow‐up. Our analyses indicated that subjects with 4 years of follow‐up had significantly higher AV45 SUVR compared to participants with only 2 years of follow‐up among the HC group (*p* = 0.025). However, there were no other significant results.

In order to investigate the association between statin duration use and dosage with baseline and longitudinal PET findings within each group, we used linear regression models. There was no significant association between statin duration use with baseline and longitudinal Aβ deposition and metabolism rate. However, statin dosage was significantly associated with Aβ deposition in 2 years (*r* = −0.412, *p* = 0.021) in the HC group. In the next step, we examined the association of total statin exposure (duration × dosage) and baseline and longitudinal PET findings. Our analysis showed a significant correlation between total statin exposure and Aβ deposition in 2‐year visit (*r* = −0.198, *p* = 0.037) in HC subjects. No associations were found in MCI and AD groups.

## 4 DISCUSSION

Our longitudinal analysis indicates that using statins might be beneficial in not increasing Aβ deposition in HC subjects. In the first 2 years of our follow‐up, no significant difference in Aβ deposition alteration was observed among healthy statin users. However, a longer follow‐up duration revealed that statins might slow down the course of Aβ deposition compared to non‐statin users. However, the within‐group analysis showed that using statin did not increase or decrease Aβ deposition. On the other hand, among MCI and AD subjects, there is no significant difference in baseline and longitudinal Aβ deposition between statin users and non‐users. In other words, we found that once the cognitive impairment begins, statin therapy fails to slow down the aggregation of Aβ and might not be beneficial for patients with cognitive impairment. Thus, earlier intervention is necessary because Aβ deposition starts decades prior to developing clinical symptoms.

Cholesterol dysregulation has been shown to affect Aβ metabolism at various stages, including fibrillation, breakdown, and transportation, into CNS cells.^20^ It is suggested that the cholesterol in the lipid bilayer facilitates early aggregation of Aβ.^21^ Similarly, free cholesterol accelerates Aβ aggregation, as the number and size of Aβ aggregates generated in the presence of free cholesterol are larger than those formed in the absence of free cholesterol.^22^ As with other proteins, the amount of Aβ depends on the balance between its production and clearance. Blood vessels of the brain play a crucial role in controlling Aβ clearance.^23^ LRP1, an apoE receptor, may be involved in the clearance of Aβ via blood vessels.^24^ Statins promote clearance of Aβ by up‐regulating the amount of LRP1 in the vessels.^25^ ApoE, one of the brain's primary cholesterol transporters, is the most potent genetic risk factor associated with AD.^26^ In both AD patients and cognitively normal individuals, the APOE‐ε4 allele promotes the deposition of senile plaques.^27^

Furthermore, improved vascular health and blood flow are linked to lower cholesterol levels. Pathologically damaged vessels have been observed within the brain of AD patients.^23^ Several studies have demonstrated that statins reduce plasma cholesterol levels and prevent cerebrovascular and cardiovascular events.^28^Thus, in combination with antihypertensive drugs that protect vessels, statins can reduce the risk of vascular dementia.^29^

Statins have also been demonstrated to affect brain cells independent of cholesterol metabolism, affecting neurotransmitter levels, synaptic neurotransmitter receptors, cellular survival, neuronal dendritic arborization, and myelination.^30^ For instance, a study of animal design found that statins altered gene expression in the brain cortex of mice and that the impacted genetic pathways were primarily connected to apoptotic mechanisms.^31^ Furthermore, in vitro studies have proven that statins promote Aβ clearance by up‐regulating insulin‐degrading enzymes, which degrades Aβ.^32^ Moreover, it has been suggested that the anti‐inflammatory effects of statins may play a significant role in preserving neuronal health.^33^ A combination of these findings indicates that statin effects, particularly those on cholesterol metabolism, can be regarded as a drug target for preventing and controlling AD.^34^ However, our findings suggest a preventive role for statins and did not support the therapeutic effects as there was no difference in Aβ changes among patients with cognitive impairment (AD and MCI).

In recent years, PET imaging, particularly with FDG and AV45 tracers, has become an essential tool in the early detection of AD biomarkers, including Aβ deposition and hypometabolism.^35^ FDG PET scan measures glucose consumption in brain regions and is an early biomarker of brain hypoactivity and hypometabolism in neurodegenerative diseases.^36^ On the other hand, the AV‐45 PET scan technique is an amyloid scan that visualizes amyloid aggregation and plaque formation in AD with high sensitivity.Thus, amyloid PET scan SUVR is accepted as a highly sensitive early‐phase predictor for mild stages of AD.^37^

It is widely accepted that the therapeutic and preventive effects of statins on AD should be studied separately.^13^ Our results demonstrated no significant difference in Aβ deposition between statin users and non‐users among AD patients. Similarly, most of the previous studies focusing on statins as a medical solution to improve cognition in AD patients have failed to determine any significant effects.^38^ However, a meta‐analysis of studies on AD patients suggests that statin consumption may slightly slow cognitive impairment in mild‐to‐moderate dementia.^12^ Based on our findings, using statins seems to slow down and stabilize the Aβ pathology due to the aging process in healthy individuals compared to non‐users. However, the within‐group analysis showed that there was no increase or decrease in Aβ deposition due to the statin use. Furthermore, several studies have determined the protective effects of statins in preventing AD in healthy individuals.^39^ In line with our study, a meta‐analysis has revealed that only long‐term use of statins can reduce the incidence of AD.^39^ Furthermore, the protective effects of statins were more prominent in younger subjects, implying that statin use should begin early in midlife to slow down or reduce the risk of AD.^13^ A study by Jeong et al. revealed that an increased risk of AD is associated with less persistent statin use, while there is decreased risk of AD in whom persistently use statins.^40^ Overall, current literature and our findings demonstrated that statins could be a preventing agent, which can slow down AD pathology and decrease the risk of dementia, but we cannot imagine a role for statin drugs in the treatment of AD.^41^

While our statin users had more than 6 years of history of drug use, we observed a significant difference in Aβ deposition alteration only after 4 years of follow‐up. Since we do have not imaging data of participants at the beginning of the statin use, it is not clear how much time is required to observe the preventive effect of statins on Aβ deposition. However, according to our study, there was no association between statin duration use with Aβ deposition and metabolism rate. Future studies should investigate the effect of statin duration use on Aβ deposition with longer follow‐up to capture the possible long‐term Aβ level alterations.

We found a significant correlation between statin dosage and Aβ deposition during the follow‐up. We can conclude that the higher dosage of statin use is more important than the longer duration use of the statins in order to prevent Aβ and slow down AD. A previous study reported that every 5‐mg increase in the daily dose of statin use was associated with reducing 11% dementia risk.^10^ However, further studies are needed to confirm these results in an epidemiological manner. Also, an investigation of the effect of each type of statins on the Aβ pathology and risk of AD should be performed.

In addition to cognitive scores, several imaging and laboratory markers have been utilized in earlier studies to investigate the effect of statins on pathophysiological mechanisms responsible for neurodegeneration. Investigations have identified altered AD biomarkers in CSF among statin users compared to non‐users, implying that statins may affect Aβ and tau protein metabolism.^42^ However, the imaging findings are controversial. A longitudinal clinical trial investigated the effect of statins on white matter microstructure and volume and indicated that long‐term statin use has a protective effect.^43^ In contrast, in a recent multimodal cross‐sectional study that utilized diffusion tensor imaging (DTI) for assessing white matter integrity along with an amyloid PET scan for assessing amyloid plaque formation, statin usage was associated with worse integrity of the white matter in the genus of the corpus callosum. In contrast, no significant alteration was observed in amyloid PET scan findings.^44^ The white matter findings can be explained by underlying vascular problems that might pre‐exist in statin users. In comparison, our longitudinal study revealed significant changes in Aβ deposition and suggested that longitudinal studies are necessary to assess the effect of statins on brain Aβ changes.

Differences in the result of the existing studies can be explained mainly by different kinds of statins, ethnicity, race, gender, and genetic differences among the study participants. Lipophilic statins, including atorvastatin and simvastatin, provide a more substantial protective effect among different statin types, owing to better blood–brain barrier penetration.^12^, ^45^ Simvastatin, the most commonly consumed statin in our cohort, had the most neuroprotective effects based on a recent in vitro study of several potential factors affecting neurodegenerative mechanisms.^45^

### 4.1 Limitations and strengths

The limitations of our study are as follows: first, this is an observational study rather than a randomized clinical trial, which is required to establish causality. On the other hand, randomized clinical trials are limited by ideal conditions, highly selected populations, and short follow‐up periods. Based on the heterogeneity of the study sample, which includes those with and without a history of cardiovascular disease, our observational data are more realistic and generalizable.^46^ Second, observational data are susceptible to selection biases. One type of selection bias is confounding by indication, which could happen if statins were prescribed more frequently to those less susceptible to cognitive decline. Selection bias could also happen when some subjects are more likely to participate in research than others.^47^ Moreover, the reduced number of subjects at the follow‐up visits can significantly affect our results since overall, participants who have more healthy behaviors choose to remain in the observational study. Another limitation is that we could not compare the effects of different types of statins. Furthermore, the lack of data regarding medication compliance which can influence the results should be mentioned as a limitation. Thus, future studies are required to investigate the effect of different types of statins on Aβ deposition and metabolism. Also, we classified participants with <2 years of statin use as non‐users at baseline. In 4 year follow‐up, the classification could be changed for participants with regular statin use. Another limitation that should be discussed is the low power of analysis in 4‐year visit among patients with AD since nearly 10% of baseline patients had data in 4‐year visit. Another important limitation is that the overall AV45 SUVR difference was quite small and also, there was no significant change in Aβ deposition over follow‐up in either statin users or non‐users, which reduced the clinical significance of our primary finding (differences in rates of change).

The study has several strengths worth mentioning: Unlike most other studies, we have directly investigated the effect of statins on AD development by analyzing Aβ aggregation, rather than cognition scores that are not specific to AD and can be affected by other causes of dementia, including vascular dementia. The effect of statins on vascular health can have a resolving effect on vascular dementia and thus have a confounding effect on cognitive function scores.^48^ Therefore, in our study, Aβ deposition and brain metabolism were directly assessed as more specific ways to predict the onset of AD. Additionally, our large sample size of statin users and non‐users allows us to detect associations that otherwise would not be detected. To our knowledge, the present study is the first of its kind to investigate the longitudinal effects of chronic statin therapy on Aβ deposition and metabolism by PET findings.

## 5 CONCLUSION

The present longitudinal analysis revealed that using statins might be beneficial in slowing down or stabilizing the Aβ deposition due to aging in subjects without cognitive impairment. However, once the clinical symptoms of cognitive impairment appear, statins fail to slow down Aβ deposition and make any difference. Moreover, the within‐group analysis showed no significant changes in Aβ among statin users and non‐users. In other words, statins seem to be preventive rather than a therapeutic agent. In contrast, statins failed to affect cerebral metabolism during our follow‐up. Overall, our findings revealed that statin users might have slower Aβ aggregation compared to non‐users. However, further studies are required to confirm our findings.

## CONFLICT OF INTEREST STATEMENT

The author declares no conflict of interest regarding the publication of this paper.

## CONSENT FOR PUBLICATION

This manuscript has been approved for publication by all authors.

## Data Availability

The datasets analyzed during the current study are available upon request with no restriction.
